# Computational framework for targeted high-coverage sequencing based NIPT

**DOI:** 10.1371/journal.pone.0209139

**Published:** 2019-07-08

**Authors:** Hindrek Teder, Priit Paluoja, Kadri Rekker, Andres Salumets, Kaarel Krjutškov, Priit Palta

**Affiliations:** 1 Competence Centre on Health Technologies, Tartu, Estonia; 2 Institute of Biomedicine and Translational Medicine, Department of Biomedicine, University of Tartu, Tartu, Estonia; 3 Institute of Computer Science, University of Tartu, Tartu, Estonia; 4 Institute of Clinical Medicine, Department of Obstetrics and Gynecology, University of Tartu, Tartu, Estonia; 5 Department of Obstetrics and Gynecology, University of Helsinki and Helsinki University Hospital, Helsinki, Finland; 6 Research Program of Molecular Neurology, Research Programs Unit, University of Helsinki and Folkhälsan Institute of Genetics, Helsinki, Finland; 7 Estonian Genome Center, Institute of Genomics, University of Tartu, Tartu, Estonia; 8 Institute for Molecular Medicine Finland (FIMM), University of Helsinki, Helsinki, Finland; Illumina Inc, UNITED STATES

## Abstract

Non-invasive prenatal testing (NIPT) enables accurate detection of fetal chromosomal trisomies. The majority of publicly available computational methods for sequencing-based NIPT analyses rely on low-coverage whole-genome sequencing (WGS) data and are not applicable for targeted high-coverage sequencing data from cell-free DNA samples. Here, we present a novel computational framework for a targeted high-coverage sequencing-based NIPT analysis. The developed framework uses a hidden Markov model (HMM) in conjunction with a supplemental machine learning model, such as decision tree (DT) or support vector machine (SVM), to detect fetal trisomy and parental origin of additional fetal chromosomes. These models were developed using simulated datasets covering a wide range of biologically relevant scenarios with various chromosomal quantities, parental origins of extra chromosomes, fetal DNA fractions, and sequencing read depths. Developed models were tested on simulated and experimental targeted sequencing datasets. Consequently, we determined the functional feasibility and limitations of each proposed approach and demonstrated that read count-based HMM achieved the best overall classification accuracy of 0.89 for detecting fetal euploidies and trisomies on simulated dataset. Furthermore, we show that by using the DT and SVM on the HMM classification results, it was possible to increase the final trisomy classification accuracy to 0.98 and 0.99, respectively. We demonstrate that read count and allelic ratio-based models can achieve a high accuracy (up to 0.98) for detecting fetal trisomy even if the fetal fraction is as low as 2%. Currently, existing commercial NIPT analysis requires at least 4% of fetal fraction, which can be possibly a challenge in case of early gestational age (<10 weeks) or high maternal body mass index (>35 kg/m^2^). More accurate detection can be achieved at higher sequencing depth using HMM in conjunction with supplemental models, which significantly improve the trisomy detection especially in borderline scenarios (e.g., very low fetal fraction) and enables to perform NIPT even earlier than 10 weeks of pregnancy.

## Introduction

It is well known that chromosomal aneuploidies are the leading cause of spontaneous miscarriages and congenital disorders in humans [1,2]. At least 10% of all clinically confirmed pregnancies are aneuploid [3]. It is assumed that the majority of fetuses with chromosomal abnormalities are spontaneously aborted during the earliest stages of pregnancy [3]. The most common aneuploidies are trisomies, which are characterized by the presence of an additional chromosome and caused by segregation errors, mainly occurring during meiotic divisions. In the case of chromosome 21 trisomy, approximately 5% of extra chromosomes have been determined to be of paternal origin and more than 90% are of maternal origin [4–9]. Despite routinely performed prenatal screenings in most developed countries, more than 0.1% of all live births are trisomic and the corresponding risk continues to rise with increasing maternal age [10].

Advanced non-invasive methods for prenatal screening using cell-free DNA (cfDNA) have considerably improved the detection of fetal aneuploidies [11]. The most commonly used technique, whole-genome sequencing (WGS) based non-invasive prenatal testing (NIPT) enables to estimate the ploidy of each chromosome by counting the sequencing reads aligned to each chromosome [12,13]. Although NIPT offers increased accuracy compared to the first trimester serum screening and ultrasound, due to its high cost, it is usually not part of the conventional prenatal screening in the majority of countries. Due to the latter and also to overcome some technical challenges (*e*.*g*. high uncertainty in trisomy detection in case of low fetal fraction), WGS-based NIPT related experimental and computational methods are continuously being developed and improved [14].

Moreover, alternative NIPT techniques also have the potential to reduce the cost related limitations of NIPT, for example by using targeted sequencing approach [15–17]. Instead of low-coverage WGS-based NIPT, only certain genomic regions are analyzed at high coverage. Targeting involves the use of hybridization-based capture or multiplex PCR amplification to enrich the genomic regions of interest [15,16]. Compared to the WGS-based methods, targeted approaches require less cfDNA, enable to study more samples in parallel and can enable confident interrogation of smaller relevant genetic markers (e.g. known microdeletion loci), making it possibly a cost-efficient alternative or complementary method [17]. A few already available targeted solutions rely on sequencing single nucleotide polymorphisms (SNPs). In these cases, the allelic distribution of heterozygous SNPs serves as an extra source of information for inferring fetal aneuploidies [18]. For example, NATUS software, developed by Natera, Inc., considers parental genotypes and crossover frequency data to calculate the expected allele distributions for SNPs and possible fetal genotypes based on recombination sites in the parental chromosomes [19]. The algorithm compares predicted allelic distributions with measured allelic distributions by employing a Bayesian-based maximum likelihood approach to determine the relative likelihood of chromosomal copy number hypothesis. The likelihoods of each sub-hypothesis are summarized and the hypothesis with the maximum likelihood is the chromosome copy number in the fetal DNA fraction (FF). Although feasible, this method is proprietary and not available to the rest of the community. An alternative approach is to model a chromosome as a hidden Markov model (HMM) of sequential loci and determine the most likely chromosomal copy number status at each locus and consequently the overall chromosomal ploidy. Kermany and colleagues used HMM to detect fetal trisomy using high-density SNP markers from a trisomic individual and one parent [20], and similar HMM-based approaches have been previously used to detect both full and sub-chromosomal aneuploidies using binned read counts [21,22].

Motivated by these previously discussed challenges and recent developments in targeted sequencing methods, we aimed to develop a publicly available and open source framework for targeted (and also SNP-based) NIPT analysis. In the current study, we present a novel statistical framework for detecting fetal trisomy and possibly the parental origin of the trisomy from targeted high-coverage sequencing data of pregnant women’s cfDNA. The framework incorporates three different HMMs that utilize read counts of targeted loci, allelic ratios of targeted SNP variants, or both in combination with a decision tree (DT) or support vector machine (SVM)-based trisomy detection, without requiring any prior knowledge of parental genotypes. We provide a comprehensive evaluation of the performance and limitations of these models on simulated datasets generated for a wide range of biologically and technically relevant scenarios. These results can be used as guidelines for appropriate study design and feasibility analysis for future NIPT studies using targeted sequencing approach. Furthermore, we provide proof-of-concept results from an experimental assay with targeted sequencing dataset of normal euploid and *in vitro* generated trisomy samples that were analyzed with the read count (RC) model, demonstrating the feasibility of the developed computational framework.

## Materials and methods

### Sequencing data simulation

A total of 1,800 training and testing datasets with different conditions were generated to mimic the targeted sequencing data of pregnant women’s cfDNA samples. Simulated datasets varied in the context of (1) fetal condition–euploidy, maternally or paternally originated trisomy characteristic to meiosis I segregation failure; (2) sequencing read depth (RD)–ranging from 500 to 15,000 at increments of 500; and (3) FF–ranging from 1 to 20% at increments of 1%. Each training dataset incorporated 100 simulated chromosomes with 1,000 targeted loci for each of the conditions (for different fetal fraction, sequencing depth and fetal chromosomal condition values) to train our models. Then, we use a test dataset of 10,000 chromosomes with our models and methods to classify the target loci (and whole chromosomes) again for all the 1,800 simulated conditions, followed by the model accuracy (ACC) evaluations.

As the cfDNA of a pregnant woman contains both maternal and fetal DNA, we started the simulation with the formation of parental chromosomes. For both parents, we generated two sets of 1,000 SNPs representing a pair of homologous chromosomes. Each SNP was biallelic and both alleles had an equal likelihood of occurrence (MAF = 0.5). Before creating a fetal set of chromosomes, parental homologous chromosomes underwent a chromosomal crossover by exchanging a random number of homologous alleles. The resulting recombined chromosomes were used to form a set of fetal chromosomes according to the fetal conditions.

In addition, we generated allele counts for each SNP according to the mean sequencing coverage and FF of the dataset. One might assume that all reads in a given region would follow a Poisson distribution with a mean proportional to the copy number of the region. However, due to the various technical biases, the process is over-dispersed and the simulation distribution followed the negative binomial distribution with a variance-to-mean ratio of 3 [23]. Since spurious sequencing errors or missing data should not have considerable consequences in case of high coverage targeted sequencing data (even if not appropriately excluded by assay and platform-specific quality control procedures) and our AR and RCAR models only consider sequencing reads that are present and have the expected SNP alleles (which are known from the assay design phase), spurious sequencing errors and missing values were not considered in our simulations.

### Allelic ratio calculation

Based on the simulated data, we calculated the allelic ratio for every “informative” SNP. Only SNPs which were heterozygous in mother and/or fetus were considered as informative.

If both alleles have an equal likelihood of occurrence (MAF = 0.5), on average 75% of SNPs are informative in case of maternally originated trisomy and the proportion of informative SNPs is even higher in the case of paternally originated trisomy as both paternal alleles contributed to heterozygosity independently. The allelic ratio is defined as the number of sequencing reads carrying a major allele for a certain variant divided by the number of sequencing reads carrying a minor allele.

### Fetal fraction calculation

FF shows the proportion of cell-free fetal DNA in total cfDNA. We estimated the FF of a cfDNA sample using the allelic counts of the sample’s reference chromosome. First, we filtered the informative SNPs on the reference chromosome, where the mother was homozygous and the fetus was heterozygous (allelic ratio > 2.5). In this subset, the major allele count was the sum of maternal allele counts and 1/2 of the fetal allele count. The minor allele count was proportional to 1/2 of the fetal allele count. The FF was calculated as the median value of the ratios between 2 × minor allele counts and the sum of major and minor allele counts. The FF of a sample was calculated using the following formula:

$$FF=\text{median}\left(\frac{2\times min_{i}}{max_{i}+min_{i}}\right),$$

where FF denotes the fetal fraction, max_i_−the major allele count of SNP *i*, and min_i_−the minor allele count of SNP *i*. The median value over all informative SNPs was considered as estimated FF of a sample, which showed high similarity to actual FF (**S1 Fig**).

### Used computational classification methods

Machine learning algorithms, in general, can be considered as mathematical functions, which will take a number of values and often map it into a single value or label (e.g. certain chromosomal state). For example, given the total number of sequencing reads per studied chromosome, machine learning algorithm could map it with a label “euploidy” (as for normal diploid chromosomal euploidy) or “trisomy” (as for chromosomal trisomy), given that the algorithm has been trained before to recognize these two chromosomal states based on sequencing read data.

Hidden Markov model (HMM) works in a similar manner as the above example, but values given to HMM are no longer a number of sequencing reads per studied chromosome, but a successive sequence of values. In this example, it could be the number of sequencing reads per studied locus along a studied chromosome and the task is to assign a label (chromosomal state) “euploidy” or “trisomy” to each measurement (locus) in the sequence of studied values. While assigning a label to each locus, the HMM algorithm also considers the chromosomal state (label) of the preceding measurement (locus) and can be allowed (through a transition probability) to be either conservative or liberal in regard to assigning a state that is different from the previously assigned one. Importantly, an HMM-based implementation of chromosomal state inference can also facilitate the detection of smaller, sub-chromosomal fetal aberrations. Given that the chromosomes of interest are sufficiently covered, it is visually possible (or by using a suitable computational sub-routine) to detect longer chromosomal alterations from the plotted chromosomal state-informative data of a studied sample.

Decision tree is a supervised learning model which uses conditional control statements in a tree-like structure to classify observations into different categories. Observations follow a tree-like graph, where each internal node represents a test on a studied attribute (*e*.*g*. a number of sequencing reads) and each branch represents the outcome–assigned category–of the test. If an observation ends up in a leaf node, it is classified by the category label of that node. In this example, we used RD, FF, and the HMM state proportions of classified loci per chromosome to classify each sample into one of the three (through the *max_depth* parameter value set to 3) different categories labeled as “euploidy”, “maternal trisomy”, or “paternal trisomy”.

Support vector machine (SVM) is a supervised learning model that, given a set of training examples builds a model that is then used to assign new studied samples into one of the previously observed categories. An SVM model is a representation of a used training data set (e.g. chromosomes with different category labels like “euploidy” or “maternal trisomy” or “paternal trisomy”) as points in space, mapped in a way that samples belonging to the different underlying categories are divided by a hyperplane and clear gap around that, which is as wide as possible. New, studied samples are then mapped into that same space and based on which side of the hyperplane they fall (based on their RD, FF, HMM state proportions of classified loci per chromosome), predicted to belong to a certain category, (i.e. “euploidy”, “maternal trisomy” or “paternal trisomy”).

### Hidden Markov model

For the detection of fetal trisomy and the parental origin of the trisomy, we implemented HMM in Python (version 3.6.2) using the hmmlearn (version 0.2.0) package. First, we created three distinct models based on the observed measurements of sequential SNPs–(1) read counts (**S2A Fig**), (2) allelic ratios (**S2B Fig**), and (3) the combination of both read counts and allelic ratios (**S2B Fig**). Second, we estimated the parameters for the models empirically using the training samples. Finally, we used the Viterbi algorithm to find the most likely underlying fetal condition behind each SNP. The developed models and estimated model parameters are available at https://github.com/cchtEE/cfDNA-simulation/tree/master/models.

#### Read count model

The read count (RC) model is a 2-state HMM which enables detection of underlying fetal conditions of sequential target loci using read counts (**S2A Fig**). The possible outcome states of the model are “euploidy” and “trisomy”. The RC model is based on the hypothesis that the mean coverage of a given region is proportional to the copy number of the region. In the case of fetal trisomy, there is an extra chromosome and therefore we would expect to see a 1/2 increase in fetal read counts compared to the euploid chromosome.

#### Allelic ratio and combined models

The allelic ratio (AR) model and the combined model of read count and allelic ratio (RCAR) are both 7-state HMMs, which enable the detection of underlying fetal conditions and the parental origin of SNPs (**S2B Fig**). The AR model uses allelic ratios of sequential informative SNPs as inputs. The RCAR model incorporates sequential read counts and allelic ratios as inputs. Both models classify loci into seven categories by the allelic pattern. The allelic pattern depends on the maternal and fetal genotypes and the fetal condition (**S1 Table**). The possible outcome states of the model are “euploidy”, “trisomy”, and “paternal trisomy”. Although the “trisomy” condition includes loci typical to both maternally and paternally originated trisomy, here we associated “trisomy” with maternally originated trisomy to avoid over-estimation of paternally originated trisomy.

#### Parameter estimation

In all three HMMs, no prior distribution of the initial state was assumed. Each possible state had an equal likelihood of occurrence. The HMM transition probability was set to state-to-state stay-switch ratio of 10, which means that the observation stays 10 times more likely in the previous state than to switch to a state with a different fetal condition. The emission probabilities were obtained using the training datasets of 100 cfDNA samples with corresponding FF and sequencing coverage. In our models, the emission probabilities were approximated to a Gaussian distribution. The distribution parameters (available for all states and simulated scenarios at https://github.com/cchtEE/cfDNA-simulation/tree/master/models) were obtained for each state by calculating the mean and variance of the read counts and allelic ratios of the training samples.

### Fetal condition estimation

The chromosomal condition of a cfDNA sample was determined by the most frequently occurring underlying condition of targeted loci using the RC, AR, and RCAR models. If no condition was prevalent, the cfDNA sample was marked as unclassified.

To improve the fetal condition estimation, especially in the case of paternally originated trisomy, we applied HMM mode (the most frequently occurred state of classified loci), DT, and SVM on HMM-classified state proportions of the targeted loci. The DT and the SVM methods were implemented in Python (version 3.5.5) using scikit-learn (version 0.19.1). The DT was used with default parameters, except the maximum depth of the tree was set to three and the random state generator to 123. The SVM also used default parameters and the random state generator was set to 123. As the DT and SVM are supervised learning models, we used the training samples to fit the models. Eventually, each cfDNA sample was classified using both models by the following features–RD, FF and HMM state frequencies. The possible classification output values were identical to HMM.

To evaluate the models, we used accuracy (abbreviated as ACC). Accuracy measures how correctly the model classifies the true fetal chromosomal condition excluding a given condition. The metric can be calculated when the prevalence of fetal chromosomal condition is known as in our simulated datasets. It incorporates both specificity and sensitivity, which are often used to evaluate NIPT, not favoring one over another and is a preferred metrics as it enables a direct comparison of trisomy detection using both euploidy and trisomy samples.

### Aneuploidy detection accuracy with different number of targeted loci

In order to measure the effect of the number of targeted loci on classification accuracy, we used a subset of the simulated dataset of test samples with RD fixed to 1,000 and FF fixed to low (3%) and medium (10%) levels. Different number of targeted loci (50, 100, 200, 500, and 1,000) were sampled from each cfDNA sample of 1,000 targets and classified by the RC model. Resulting class frequencies were further classified by HMM mode, DT, and SVM. The classification accuracy of each model was measured over 10,000 cfDNA samples (**S3 Fig**).

### The MAF-dependent proportion of informative variants

First, we simulated chromosomes carrying 1000 variants, whereas at each variant locus alternative alleles were introduced randomly with the frequency of the corresponding studied minor allele frequency (MAF) of 1, 5, 10, 20, 30, 40 and 50%. Pairs of randomly selected chromosomes were combined and then, by considering two randomly selected individuals as parents, the number of resulting genotypes across the pairs of chromosomes were counted and the number of informative variants was calculated. This procedure was repeated 100 times for each MAF value and the average proportion of informative variants was calculated across these repeated simulations (**S4 Fig**). Theoretical proportion of informative variants was calculated considering unrelated parents with independent diploid chromosomes carrying variants with a given minor allele frequency.

### Experimental targeted sequencing data

We used TAC-seq targeted sequencing data of experimentally controlled trisomy samples (N = 12) as described in [17] to evaluate the RC model. In the aforementioned *in vitro* experiment, different proportions of genomic DNA from a non-trisomy 21 cell line (GM01359) were mixed with a genomic DNA from a trisomy 21 cell line (GM04616) to imitate different proportions of FF (0, 10, 20, and 100%), each in three replicas. Genomic DNA was sheared by sonication to mimic 160–180 bp cfDNA. DNA fragments were hybridized and sequenced using TAC-seq detector probes specifically designed to target only the reference chromosomes 2 (68 targeted loci) and chromosome 3 (60 targeted loci), and studied chromosome 21 (99 targeted loci). Read counts of targeted loci (mean sequencing read coverage of ~3600 across all targeted loci and samples) were converted to absolute molecular counts using unique molecular identifiers with the threshold of one to reduce the PCR amplification bias [24]. Obtained absolute molecule counts (mean coverage of ~523 across all targeted loci and samples) of each sample were filtered by interquartile range to remove outlier target loci, leaving 69 to 86 targeted loci for chromosome 21 analyses with FF of 0, 10, 20, and 100%. Resulting absolute molecule counts (with a mean coverage of ~527 reads across all loci and samples) for each target locus were used as the input data for the RC model to classify each targeted locus as “euploidy” or “trisomy”.

## Results and discussion

**Fig 1:**
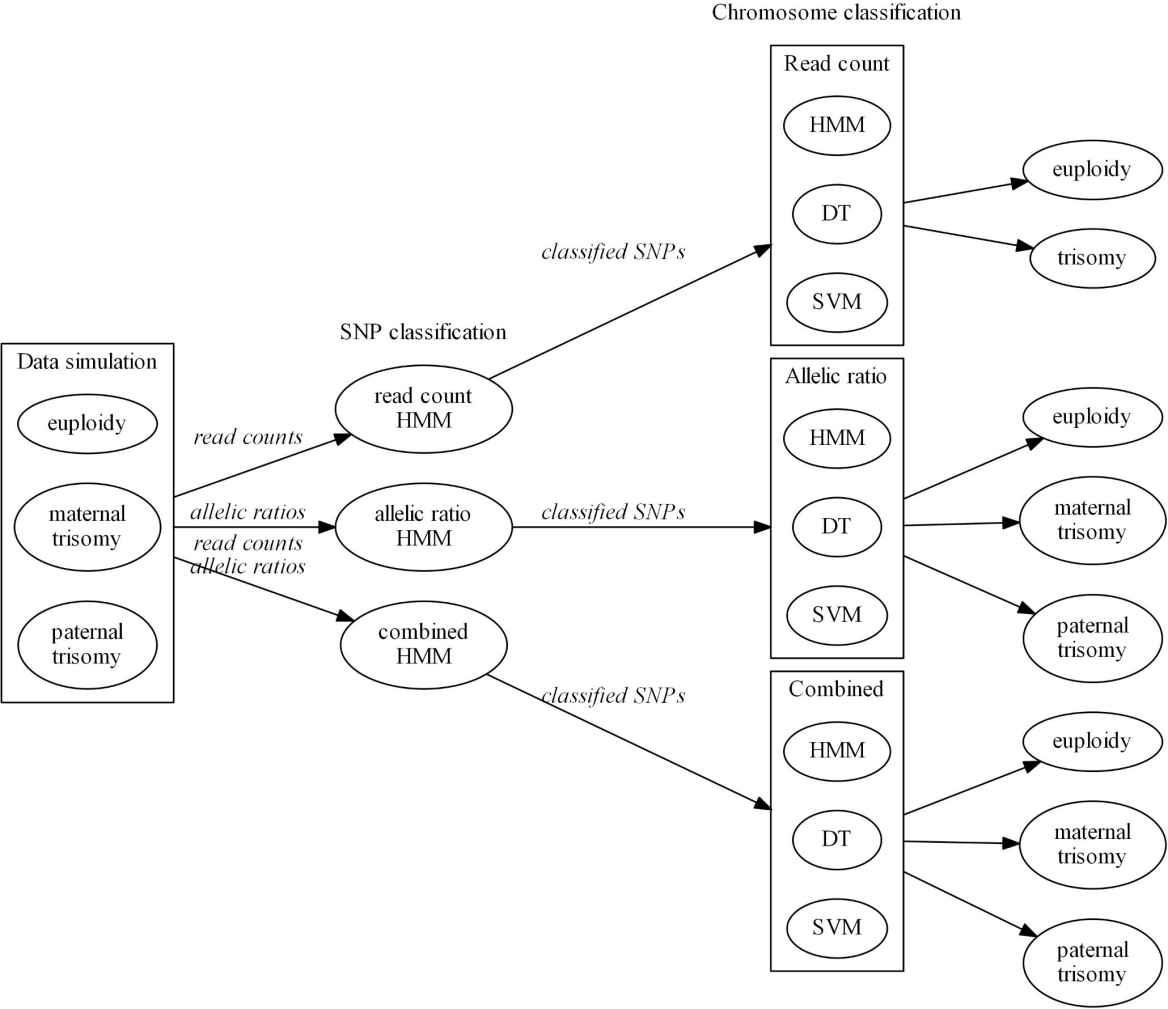
Overview of the study. The computational framework included three steps: (1) simulation of cell-free DNA samples imitating pregnancy with fetal euploidy, maternally or paternally inherited trisomy; (2) classification of loci by sample using hidden Markov model (HMM)-based read count (RC), allelic ratio (AR), and combined (RCAR) models; and (3) classification of chromosome using HMM mode, decision tree (DT), or support vector machine (SVM) on locus classification results.

We developed three novel HMM-based statistical models to detect fetal chromosomal trisomies from targeted sequencing assays. In addition to a naïve HMM-based frequentist approach for trisomy detection, we applied two machine learning (ML) methods to infer fetal trisomy (see “Used computational classification methods” in Materials and Methods). While considering a wide range of biologically and technically motivated conditions, we simulated datasets mimicking cfDNA sequencing assays and used these data to perform a comprehensive evaluation of our proposed computational methods (**Fig 1**). We also applied the RC model on targeted sequencing dataset of experimentally controlled *in vitro* trisomy samples.

### Novel HMM-based methods for trisomy detection

**Fig 2:**
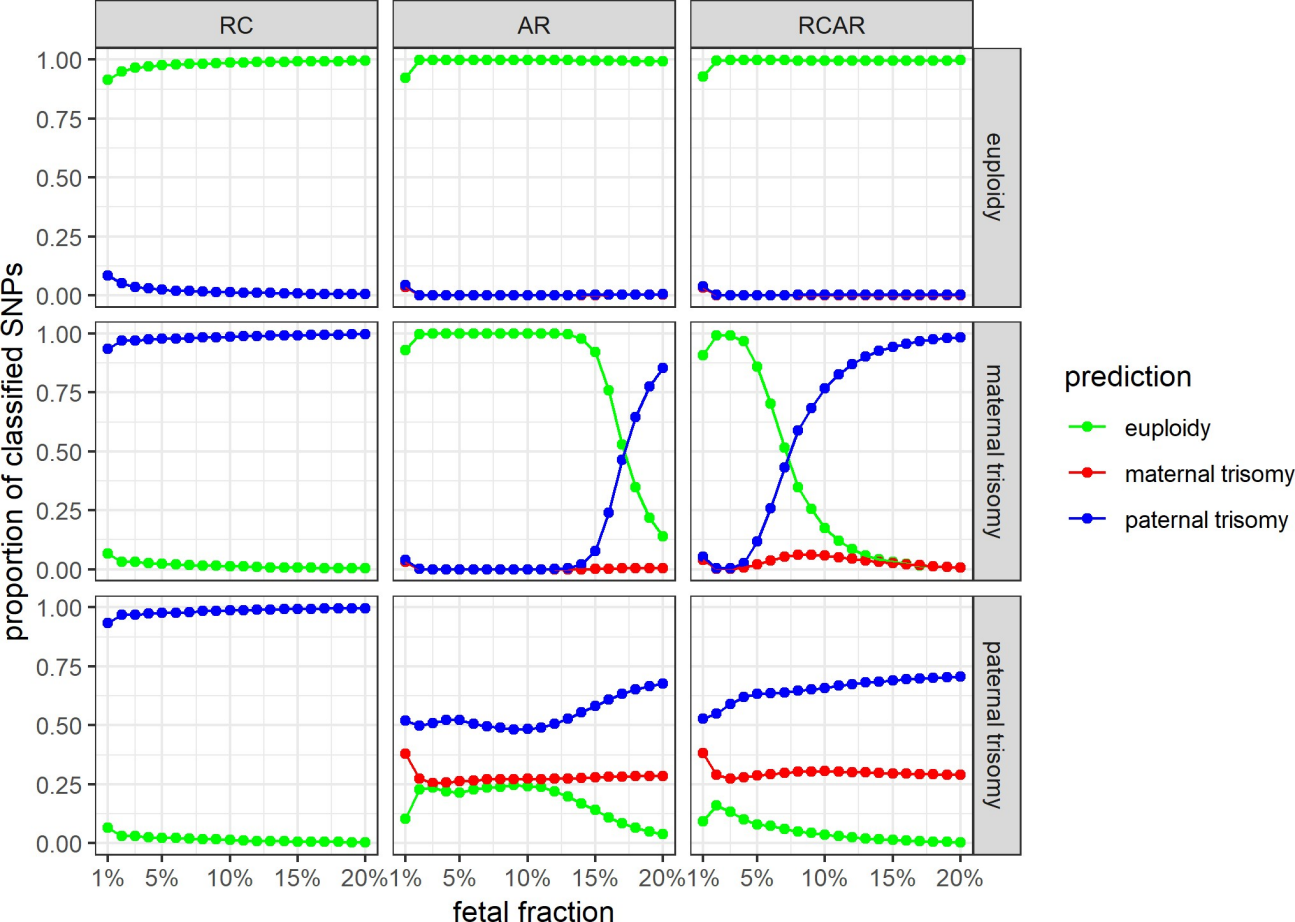
The proportion of correctly classified targets in the simulated datasets with different levels of fetal DNA fraction (FF). The simulated datasets of fetal euploidy, maternally and paternally inherited trisomy (on the horizontal panels) were classified by three hidden Markov models (on the vertical panels)–the read count (RC), the allelic ratio (AR) and the combined (RCAR) model. The sequencing read depth of a sample was fixed to a mean of 1,000 reads per reference loci. Each data point represents 10,000 cell-free DNA samples with 1,000 SNPs and the classification results for fetal euploidy, maternally and paternally inherited trisomy are represented respectively by green, red and blue color.

By considering the sequencing read counts (the RC model) of targeted loci, allelic ratios (the AR model) of targeted SNPs, or both (the RCAR model), the developed HMM models were used to classify consecutive targets on a (simulated) studied chromosome into pre-defined underlying states. In the 2-state RC model, these unique states represented fetal euploidy and trisomy (**S2A Fig**). In the case of the 7-state AR and RCAR models, these different states can occur with fetal euploidy or maternally/paternally originated trisomy (**S2B Fig**). Consequently, the proportion of loci classified into these distinct states can be used to infer the fetal condition of each studied chromosome (see “Fetal condition estimation” in Materials and Methods). And although such naïve classification works relatively well in case of high sequencing read depth (RD) and fetal fraction (FF) scenarios, the proportion of loci classified into these underlying states can be fairly similar and thus difficult to distinguish unambiguously in the case of some RD and FF ranges (**Fig 2**).

Therefore, an accurate calculation of FF is also crucial in order to control the precision and uncertainty of fetal trisomy detection and sequencing-based NIPT assays. Notably, in the case of the RC model and autosomal chromosomes there is no information that could be used to infer the FF of the studied sample so that corresponding (optimal) model parameters could be used. One possible solution to overcome this challenge is to use the expected median FF of 10% [25]. In the case of the AR and RCAR models, we used informative polymorphic SNPs with heterozygous alleles in mother and/or fetus to infer the sample-specific FF (**S1 Fig**), similarly to previous studies [26–29]. Additionally, in the case of the AR and RCAR models, allelic count data at informative SNPs can be used to calculate allelic ratios, distinguishing maternally and paternally originated trisomies (see “Allelic ratio calculation” in Materials and Methods) according to their distinct allelic patterns (**S1 Table**). On the other hand, these models only consider informative targeted SNPs that are polymorphic in a given sample, reducing the total number of analyzed SNP variants at least by 25%.

The latter was also demonstrated by theoretical and simulation-driven calculations, considering SNPs with variable minor allele frequency (**S4 Fig**). Although simplified to a certain level, these analyses demonstrate that the proportion of informative variants slowly decreases together with lower MAF of targeted variants, therefore also decreasing the trisomy detection accuracy with the AR and RCAR models. On the other hand, the proportion of informative SNP variants is relatively high (~50%, or more) down to MAF of 20% and human chromosomes carry variants with various allele frequencies. Although a relevant consideration, this can be relatively easily mitigated through an appropriate target variant selection procedure, considering the population and ethnicity-specific MAF (e.g. through data resources such as ExAC [30], gnomAD [31], or 1000 Genomes Project [32]) and minimizing the proportion of possible non-informative SNPs.

### Supplemental methods for trisomy detection

Because in case of some trisomy scenarios (such as paternally originated trisomy), the previously described HMM-based method could not unambiguously detect the underlying fetal condition (**Fig 2**), we developed two additional “supplemental” machine learning (ML)-based methods to improve trisomy classification accuracy. These supplemental methods–decision tree (DT) and support vector machine (SVM), which take HMM-classified state proportions of each studied chromosome as input and perform an enhanced stratification into fetal euploidy or trisomic states, significantly improved the sample classification accuracy (**S2 Table**). The gain in classification accuracy was more significant in situations where the proportion of loci inferred into one or the other HMM state was unambiguous (not an obvious majority) and where the naïve frequentist approach did not work (**Tables 1 and S2**).

**Table 1:** Summarized fetal euploidy and trisomy classification accuracy with different computational models and methods. Each value represents an average classification accuracy across 3,600,000 simulated cell-free DNA samples within a given range of fetal fraction, with fetal euploidy, maternally, and paternally inherited trisomy at the different sequencing depth intervals (500–15,000 reads).

Fetal fraction	RC			RC (with fixed FF)			AR			RCAR		
	HMM	DT	SVM	HMM	DT	SVM	HMM	DT	SVM	HMM	DT	SVM
1–5%	1.00	1.00	1.00	0.56	0.93	0.95	0.34	0.69	0.69	0.49	0.91	0.90
6–10%	1.00	1.00	1.00	1.00	1.00	1.00	0.54	0.92	0.92	0.65	1.00	1.00
11–15%	1.00	1.00	1.00	1.00	1.00	1.00	0.63	0.98	0.98	0.67	1.00	1.00
16–20%	1.00	1.00	1.00	1.00	1.00	1.00	0.65	0.99	0.99	0.67	1.00	1.00
total	1.00	1.00	1.00	0.89	0.98	0.99	0.54	0.89	0.90	0.62	0.98	0.98

RC–read count; AR–allelic ratio; RCAR–read count and allelic ratio; HMM–hidden Markov model; DT–decision tree; SVM–support vector machine; FF–fetal fraction

All three HMMs (RC, AR, and RCAR) independently and conjointly with the supplemental methods (DT and SVM) were tested on the same collection of simulated cfDNA datasets representing all combinations of different fetal chromosomal conditions (euploidy, maternally and paternally originated trisomy) and FFs (1–20%), sequenced with various RDs (500–15,000 reads), feasible for targeted sequencing assays.

### Read count (RC) model

**Fig 3:**
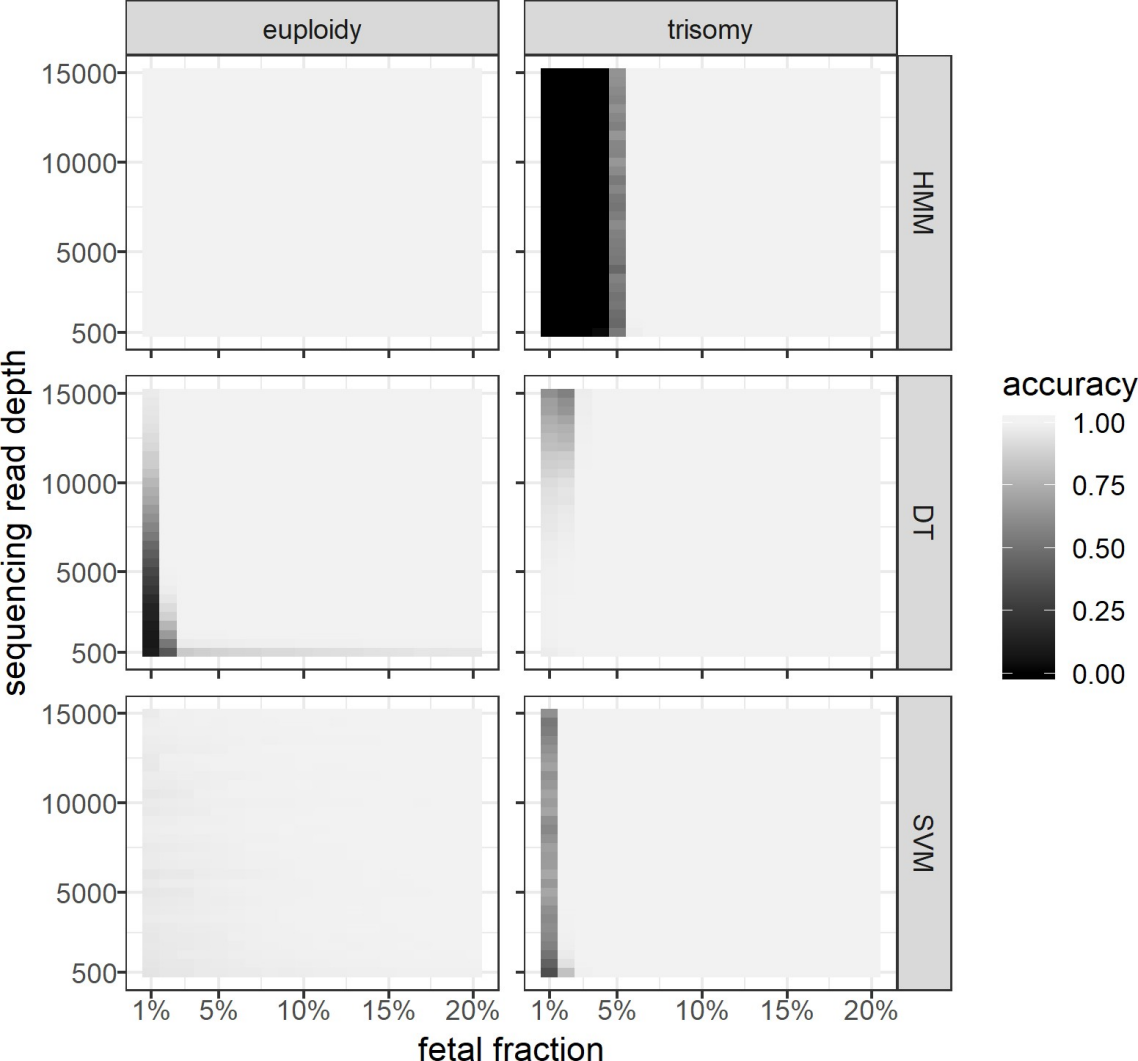
Classification accuracy of the read count (RC) model with fixed fetal fraction in conjunction with the machine learning models on simulated datasets. The simulated datasets of fetal euploidy and trisomy (vertical panels) were first classified by RC model and the resulting class frequencies were further classified by hidden Markov model (HMM) mode, decision tree (DT) and support vector machine (SVM) (horizontal panels). Each panel includes cells with different fetal DNA fractions (x-axis) and sequencing read coverages (y-axis). Each cell includes 10,000 cell-free DNA samples and the color represents the model classification accuracy.

The RC model enables detection of fetal euploidy and trisomy by using sequencing read counts in successive (targeted) regions along the chromosome of interest. As read count data alone cannot be used to infer the FF of a studied sample, we assumed FF as 10% in this testing model. Nevertheless, the HMM method showed excellent accuracy (ACC ≥ 0.99) in detecting fetal euploidy (**Fig 3**). On the other hand, this method was ineffective for detecting fetal trisomy if the FF was lower than 6% (ACC = 0.11) and increasing the RD induced only a minor increase in detection accuracy (**S2 Table**). It is also important to note that since there is no direct method to distinguish between paternally and maternally inherited alleles, the read count model does not allow determining the parental origin of trisomies. On the other hand, since it uses only sequencing read count information to detect fetal trisomies, it is straightforward to integrate this model with any targeted sequencing-based NIPT solutions.

Applying supplemental ML methods significantly improved the RC model-based classification at lower FFs (**Table 1**). The DT method allowed accurate detection of fetal euploidy and trisomy even if the FF was as low as 3%; the SVM method successfully lowered that limit even further, allowing accurate detection of fetal trisomies at FF 2%, with a small trade-off in detecting aneuploid chromosomes (**Fig 3**). Unexpectedly, DT trisomy detection improved at a lower read coverage. This can be explained by the strictly set maximum depth (*max_depth* = 3) of the DT, which prevented overfitting of the model; on the other hand, this method was not suitable for classifying a wide range of FF values. This shortcoming is due to the fixed FF parameter rather than the properties of the DT (**Figs 3 and S5**).

Additionally to the effect of different FFs and RDs, we simulated aneuploidy detection accuracy with the different number of targeted loci. These calculations demonstrate that in case of robustly working target loci (providing homogeneous sequencing coverage across the studied chromosome) and FF of 10%, only 50 target probes are required to achieve relatively high trisomy and euploidy detection accuracy (ACC ≥ 0.99) with RD of 1000 (**S3 Fig**). Furthermore, according to these simulations, as few as 200 robustly working targeted loci per chromosome of interest will provide relatively high accuracy (ACC = 0.98) for trisomy detection even with FF of 3% (**S3 Fig**).

### Allelic ratio (AR) model

**Fig 4:**
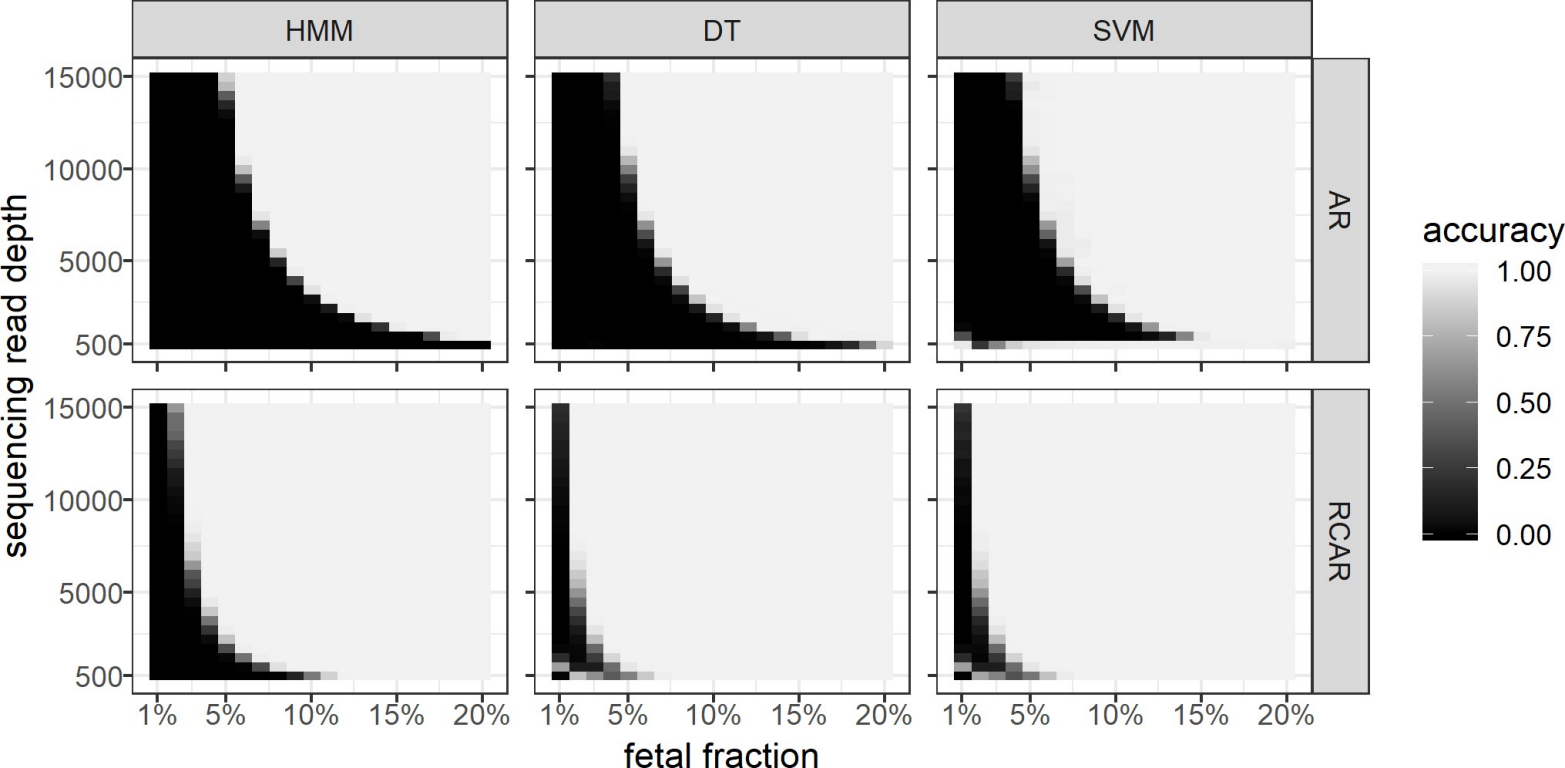
Classification accuracy of the allelic ratio (AR) and the combined (RCAR) model in conjunction with the machine learning models on the simulated dataset of maternally originated trisomy. The simulated dataset of maternally originated trisomy was first classified by the AR and RCAR models (horizontal panels) and the resulting class frequencies were further classified by hidden Markov model (HMM) mode, decision tree (DT) and support vector machine (SVM) (vertical panels). Each panel includes cells with different fetal DNA fractions (x-axis) and sequencing read coverages (y-axis). Each cell includes 10,000 cell-free DNA samples and the color represents the model classification accuracy.

The AR model uses counts of sequencing reads containing one or the other allele at informative SNP loci along the chromosome of interest to estimate if the studied sample has euploid, maternally or paternally originated trisomy and to infer the FF of the corresponding sample. The AR model showed excellent accuracy (ACC ≥ 0.99) detecting fetal euploidy even at an FF of 1% and an RD of 500 (**S6 Fig**) and reasonable accuracy to detect maternally originated trisomy if FF was ≥ 6% and RD was higher than 10,000 (**Fig 4**). In contrast to the DT and the SVM methods, it was unable to detect paternally originated trisomy in a given range of FF and RD (**S6 Fig**).

Compared to the read count data, allelic ratio information was used to estimate the FF of a sample using specific allelic patterns (**S1 Table**). In addition, allelic ratio data were used to separate maternally and paternally originated trisomies. As for the HMM, the inability to detect paternally originated trisomy can be explained by the overlapping emission distributions of the allelic ratios of maternally and paternally originated trisomies.

In general, the supplementary methods increased the detection accuracy for the AR model significantly (**Table 1**), especially in the case of paternally originated trisomy (**S2 Table and S6 Fig**). In the case of maternally originated trisomy, all three methods had similar characteristics as the detection accuracy was positively correlated with both sequencing RD and FF (**Fig 4**). The read count had a stronger impact on the AR model, whereas the RC model was mostly affected by FF. The DT had a slight fetal trisomy detection improvement compared to the HMM, and the SVM, in turn, had a slight advantage over the DT. The DT methods also showed very high accuracy (ACC ≥ 0.99) in detecting fetal euploidy. Unlike the other methods, the SVM showed slightly better maternally originated trisomy detection accuracy (ACC = 0.71) and consistently good results (ACC = 0.92) if the read coverage was low (RD = 500); on the other hand, the SVM had poor results (ACC = 0.27) detecting fetal euploidy if the read coverage was low (RD = 500). The SVM’s failure in case of euploidy and excellent results for maternally originated trisomy at low read coverage contradicted each other, which was a sign of maternally originated trisomy over-estimation. In the case of paternally originated trisomy, the DT and SVM had excellent (ACC ≥ 0.99) detection accuracy (**S2 Table**).

### Combined (RCAR) model

Finally, we studied the RCAR model, which incorporates both read count and allelic ratio information to predict fetal euploidy or trisomy. Furthermore, it utilizes informative SNPs, which enables separation of maternally and paternally originated trisomy by allelic patterns (**S1 Table**) and estimated FF. The RCAR model showed excellent results (ACC ≥ 0.99) in detecting fetal euploidy (**S7 Fig**). Compared to the HMM, the supplemental methods were inefficient (ACC < 0.83) to detect fetal euploidy when the FF and read coverage was low (RD ≤ 1,500; FF ≤ 3%). All three methods showed a positive correlation between detection accuracy, RD and FF, while the HMM detection accuracy was substantially lower (ACC = 0.62) as compared to the supplemental methods (ACC = 0.98). In the case of maternally originated trisomy, the DT and the SVM had better detection accuracy (ACC = 0.93) than HMM (**Fig 4**). In the case of paternally originated trisomy, the DT had excellent detection accuracy (ACC ≥ 0.99) followed closely by the SVM (**S2 Table**). However, the HMM was unable to detect paternally originated trisomy in any give range of FF and read coverage (**S7 Fig**).

The RCAR model showed significantly higher accuracy in conjunction with supplemental methods (**Table 1**). Compared to the HMM, the supplementary methods increased the detection accuracy in the case of fetal trisomies (**S7 Fig**). As for the HMM, the inability to detect paternally originated trisomy can be explained by the overlapping emission distributions (allelic ratios) of maternally and paternally originated trisomy. Similarly to the AR model, the overall accuracy of the RCAR model was affected by both FF and sequencing RD, whereas the RC model was mostly affected by FF (**Figs 3 and S5**).

### Experimental targeted sequencing data

**Fig 5:**
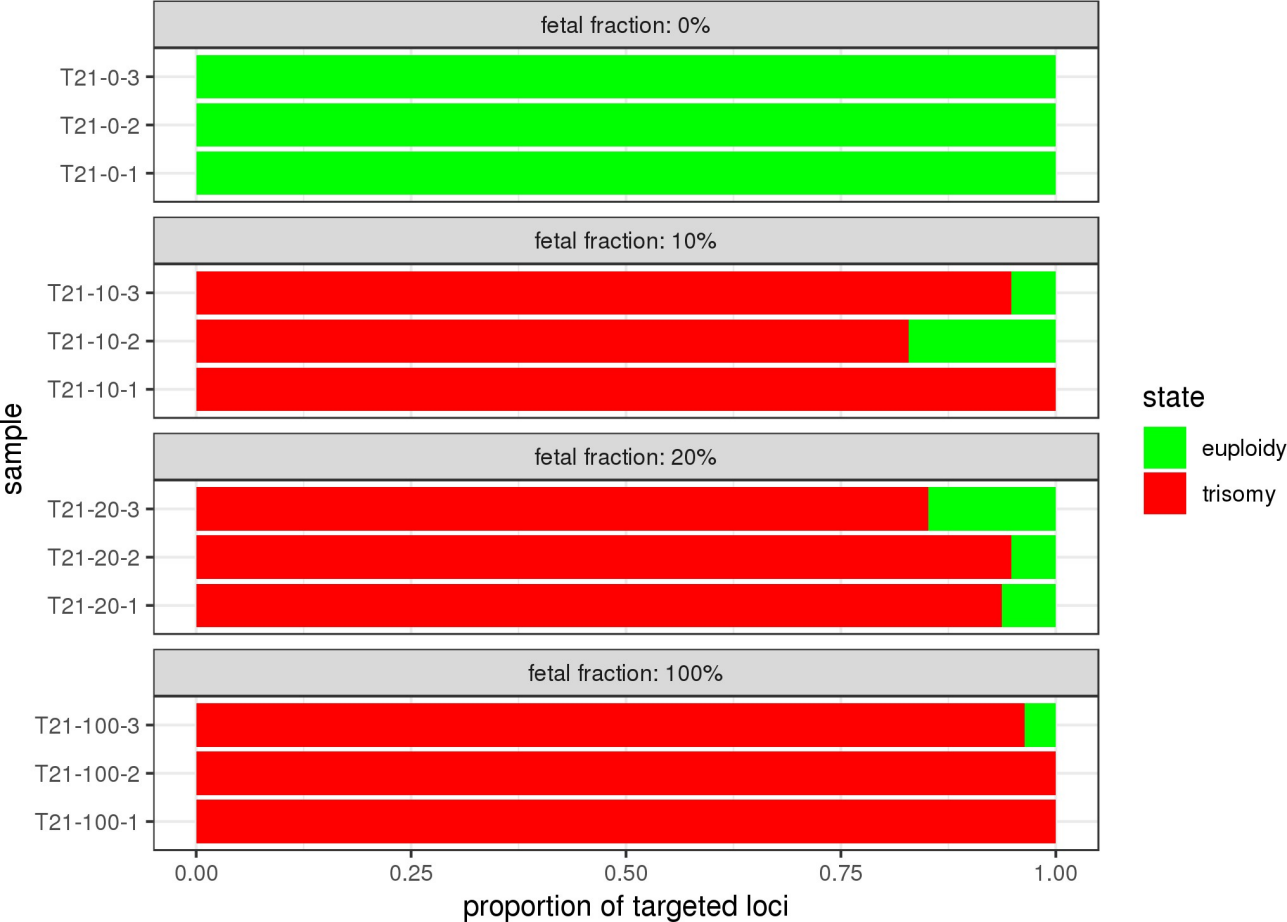
Classification of targeted loci using the read count (RC) model on experimental targeted sequencing data. Experimentally controlled *in vitro* trisomy samples were created by mixing different proportions of genomic DNA from a non-trisomy 21 cell line with a genomic DNA from a trisomy 21 cell line to imitate different proportions of FF (horizontal panels), each in three replicas. Genomic DNA was sheared by sonication to mimic 160–180 bp cfDNA. DNA fragments were hybridized and sequenced using TAC-seq detector probes specifically designed to target only the reference chromosome 2 (68 targeted loci) and chromosome 3 (60 targeted loci), and the studied chromosome 21 (99 targeted loci). Read counts of targeted loci were converted to absolute molecular counts using unique molecular identifiers with the threshold of one to reduce the PCR amplification bias. Obtained absolute molecule counts of each sample were filtered by interquartile range to remove outliers and used as input to RC model to classify each targeted locus as “euploidy” (green color) or “trisomy” (red color). Proportions of classified loci per chromosome per sample are visualized as horizontal bars.

To evaluate our HMM model with experimental targeted sequencing data, we designed a simple proof-of-concept experiment by using previously published and newly generated targeted sequencing data (see “Experimental targeted sequencing data” in Materials and Methods). We applied the RC model on the absolute molecule counts of targeted loci on chromosome 21 to categorize consecutive target loci to fetal euploidy and trisomy (**Fig 5**). The results of these analyses demonstrate the feasibility of our approach even with a relatively low sequencing coverage (on average, 527 reads per target locus in each sample) and a small number of targeted loci (from 69 up to 86 after locus-wise quality control procedures) per studied chromosome. This experiment also demonstrated the necessity of robustly working target probes (each probe interrogating a specific intended locus) and sequencing assay, providing homogeneous sequencing coverage across the studied chromosome. Otherwise, target loci with systematically lower (or possibly higher) sequencing coverage can be consequently in some occasions classified to the wrong chromosomal state (**S8 Fig**).

The next step with any actual targeted sequencing assay and platform (in conjunction with our computational methods) would be its experimental testing (followed by assay/target set optimization, *e*.*g*. to replace/exclude incorrectly reporting targets) and finally, depending on its intended use, (clinical) validation with an independent set of samples from pregnant women, including sensitivity and specificity estimates for different targeted chromosomes.

## Conclusions

Targeted sequencing approaches have the potential to reduce the price of NIPT and improve the quality of healthcare. In the current study, we present HMM-based models in conjunction with supplemental methods (DT and SVM), which enabled the detection of fetal trisomy and the parental origin of an extra chromosome using targeted sequencing-based prenatal (NIPT) assays. The developed methods were tested on simulated datasets generated for a wide range of biologically and technically motivated scenarios to determine the functional feasibility and limitations of each approach.

We determined that regardless of the computational method used, the most challenging factor in fetal trisomy detection is low FF. In our study, the RC model in conjunction with ML-based supplemental methods can detect fetal trisomy at 2% FF, which in turn enables earlier confident NIPT testing. Although the RC model can be easily incorporated into all targeted sequencing based NIPT workflows, the RCAR model is the recommended choice in case of SNP-based targeted assays for its high accuracy and ability to determine the parental origin of the trisomy and to accurately estimate the studied sample FF.

We provide a proof-of-concept results from an experimental assay with targeted sequencing dataset, demonstrating the feasibility of our models and the developed computational framework. As the computational framework and methods presented in the current study are sequencing platform and assay agnostic, these are in principle usable for a wide selection of different existing and future targeted NIPT assays and platforms.

## Supporting information

S1 Fig Difference between estimated and simulated fetal fraction (FF). The simulated FF was subtracted from the estimated FF for each simulated cell-free DNA sample to determine the FF difference (y-axis). The differences were grouped as boxplots by sequencing read depth (x-axis). The results show a positive correlation between sequencing read depth and FF estimation accuracy.
(TIF)

S2 Fig The architecture of 2- and 7-state hidden Markov models (HMMs). (A) The 2-state HMM classified sequential single nucleotide polymorphisms (SNPs) into 2 underlying states, which represent fetal euploidy (white) and trisomy (grey), using read counts. (B) The 7-state HMM classified SNPs into 7 underlying states, which represent fetal euploidy (white), maternally (white-grey) and paternally originated trisomy (grey-white), using allelic ratios with or without read counts.
(TIF)

S3 Fig Classification accuracy of the read count (RC) model in conjunction with the machine learning models on simulated datasets depending on the number of targeted loci. The simulated datasets of fetal euploidy and trisomy (horizontal panels) were first classified by RC model and the resulting class frequencies were further classified by the hidden Markov model (HMM) mode, decision tree (DT) and support vector machine (SVM) at 3% and 10% fetal fraction (vertical panels). The sequencing read depth of a sample was fixed to a mean of 1,000 reads per reference loci. Each data point represents 10,000 cell-free DNA samples. The classification accuracy of each model was measured with the different number of targeted loci (x-axis).
(TIF)

S4 Fig The proportion of informative SNP variants in case of the different minor allele frequencies (MAFs). The proportion of informative variants (y-axis) in case of MAF of 1, 5, 10, 20, 30, 40 and 50% (x-axis) in 100 simulations with each MAF are represented with the box plots. The theoretical proportion of informative variants is denoted next to corresponding box plots for each MAF.
(TIF)

S5 Fig Classification accuracy of the read count (RC) model in conjunction with the machine learning models on simulated datasets. The simulated datasets of fetal euploidy and trisomy (vertical panels) were first classified by RC model and the resulting class frequencies were further classified by hidden Markov model (HMM) mode, decision tree (DT) and support vector machine (SVM) (horizontal panels). Each panel includes cells with different fetal DNA fractions (x-axis) and sequencing read coverages (y-axis). Each cell includes 10,000 cell-free DNA samples and the color represents the model classification accuracy.
(TIF)

S6 Fig Classification accuracy of the allelic ratio (AR) model in conjunction with the machine learning models on simulated datasets. The simulated datasets of fetal euploidy, maternally and paternally trisomy (vertical panels) were first classified by the AR model and the resulting class frequencies were further classified by hidden Markov model (HMM) mode, decision tree (DT) and support vector machine (SVM) (horizontal panels). Each panel includes cells with different fetal DNA fractions (x-axis) and sequencing read coverages (y-axis). Each cell includes 10,000 cell-free DNA samples and the color represents the model classification accuracy.
(TIF)

S7 Fig Classification accuracy of the combined (RCAR) model in conjunction with the machine learning models on simulated datasets. The simulated datasets of fetal euploidy, maternally and paternally trisomy (vertical panels) were first classified by RCAR model and resulting class frequencies were further classified by hidden Markov model (HMM) mode, decision tree (DT) and support vector machine (SVM) (horizontal panels). Each panel includes cells with different fetal DNA fractions (x-axis) and sequencing read coverages (y-axis). Each cell includes 10,000 cell-free DNA samples and the color represents the model classification accuracy.
(TIF)

S8 Fig Classification of targeted loci using the read count (RC) model on experimental targeted sequencing dataset. Experimentally controlled *in vitro* trisomy samples were created by mixing different proportions of genomic DNA from a non-trisomy 21 cell line with a genomic DNA from a trisomy 21 cell line to imitate different proportions of FF (horizontal panels), each in three replicas. Genomic DNA was sheared by sonication to mimic 160–180 bp cfDNA. DNA fragments were hybridized and sequenced using TAC-seq detector probes specifically designed to target only the reference chromosome 2 (68 targeted loci) and chromosome 3 (60 targeted loci), and the studied chromosome 21 (99 targeted loci). Read counts of targeted loci were converted to absolute molecular counts using unique molecular identifiers with the threshold of one to reduce the PCR amplification bias. Obtained absolute molecule counts of each sample were filtered by interquartile range to remove outliers and used as input to RC model to classify each targeted locus as “euploidy” (green color) or “trisomy” (red color). Sequentially classified targeted loci per sample are visualized as colored dots.
(TIF)

S1 Table Allelic patterns. Allelic ratio depends on fetal chromosomal condition, and maternal and fetal genotype.
(DOCX)

S2 Table Fetal trisomy classification accuracy with different computational models and methods. Each value represents an average classification accuracy over 1,200,000 simulated cell-free DNA samples with fetal (maternally or paternally inherited) trisomy at the different sequencing depth intervals (500–15,000 reads).
(DOCX)
