# Supplementary figures and images for: Computational framework for targeted high-coverage sequencing based NIPT
Source: PLoS One. 2019 Jul 8;14(7):e0209139. doi: 10.1371/journal.pone.0209139 (PMC6613673; doi:10.1371/journal.pone.0209139)

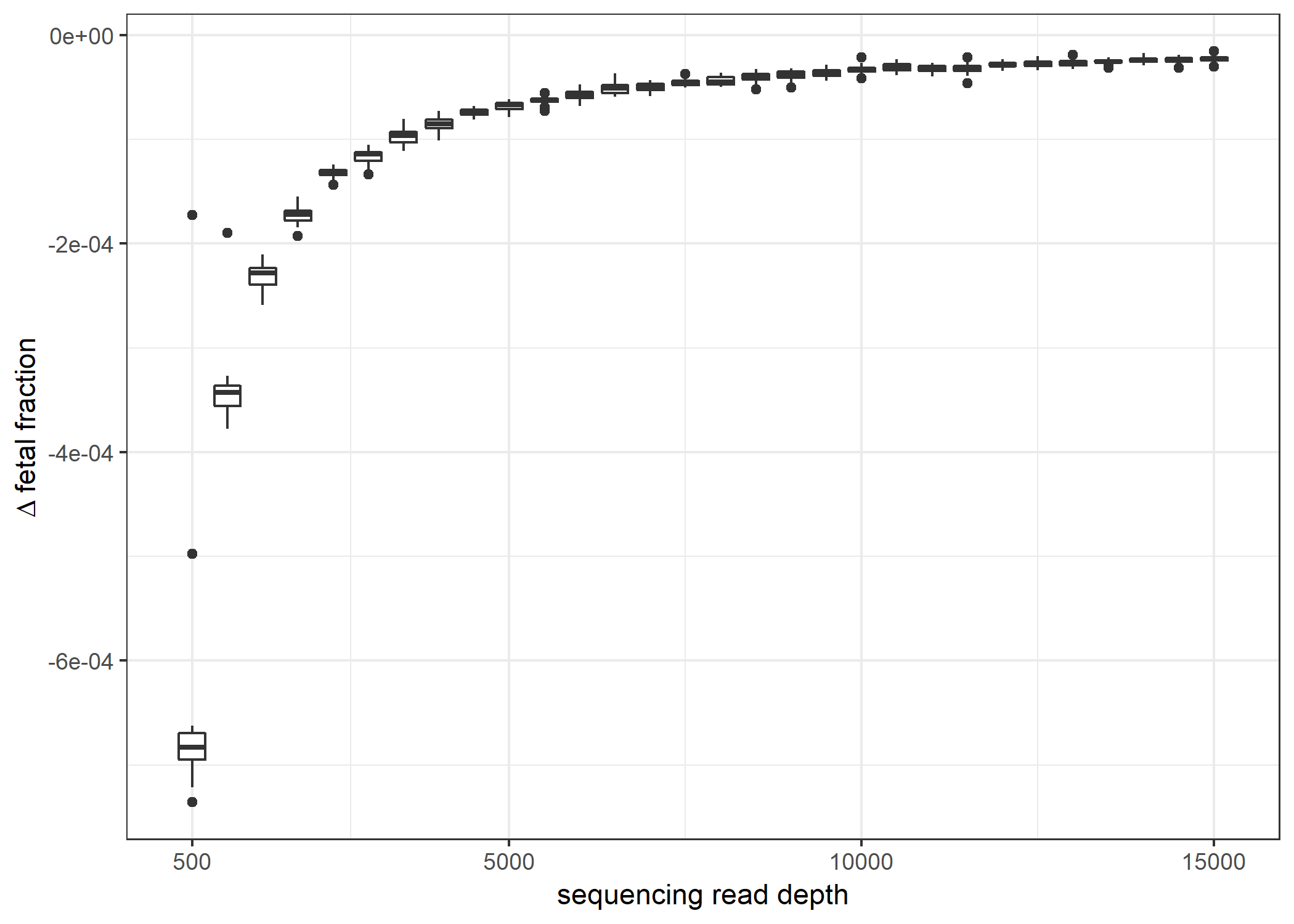

Supplement: S1 Fig — The simulated FF was subtracted from the estimated FF for each simulated cell-free DNA sample to determine the FF difference (y-axis). The differences were grouped as boxplots by sequencing read depth (x-axis). The results show a positive correlation between sequencing read depth and FF estimation accuracy. (TIF) [file pone.0209139.s001.tif]

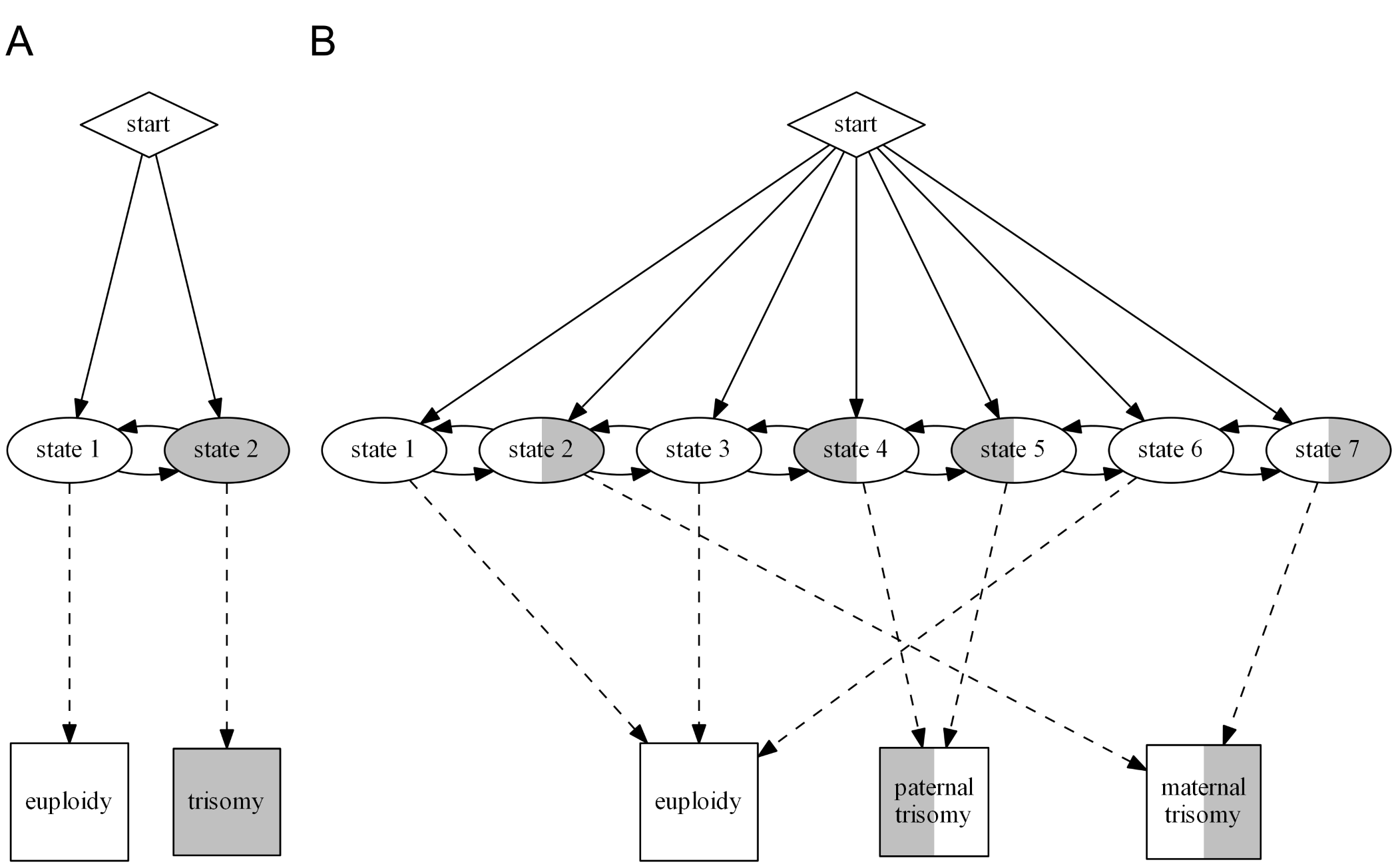

Supplement: S2 Fig — (A) The 2-state HMM classified sequential single nucleotide polymorphisms (SNPs) into 2 underlying states, which represent fetal euploidy (white) and trisomy (grey), using read counts. (B) The 7-state HMM classified SNPs into 7 underlying states, which represent fetal euploidy (white), maternally (white-grey) and paternally originated trisomy (grey-white), using allelic ratios with or without read counts. (TIF) [file pone.0209139.s002.tif]

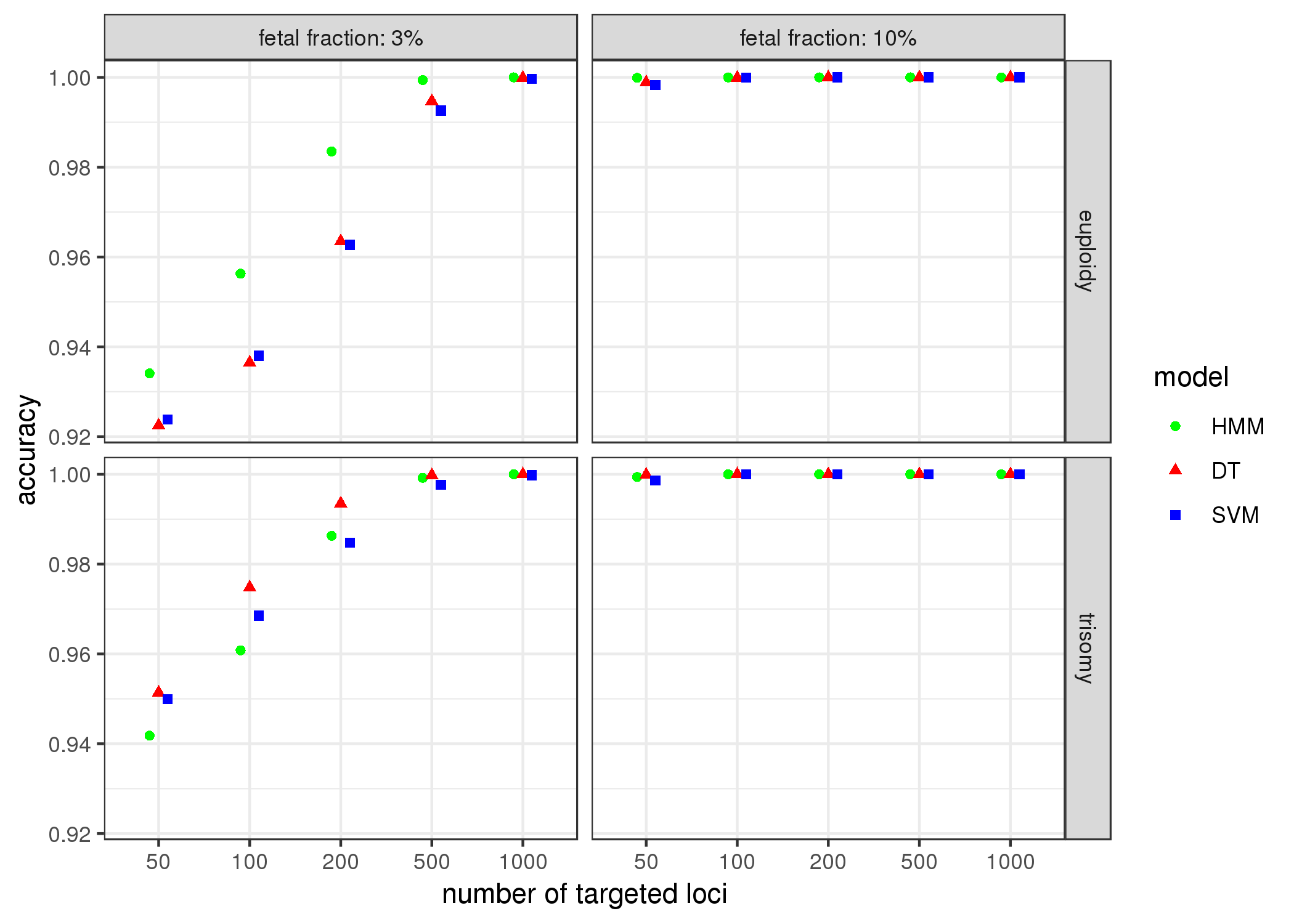

Supplement: S3 Fig — The simulated datasets of fetal euploidy and trisomy (horizontal panels) were first classified by RC model and the resulting class frequencies were further classified by the hidden Markov model (HMM) mode, decision tree (DT) and support vector machine (SVM) at 3% and 10% fetal fraction (vertical panels). The sequencing read depth of a sample was fixed to a mean of 1,000 reads per reference loci. Each data point represents 10,000 cell-free DNA samples. The classification accuracy of each model was measured with the different number of targeted loci (x-axis). (TIF) [file pone.0209139.s003.tif]

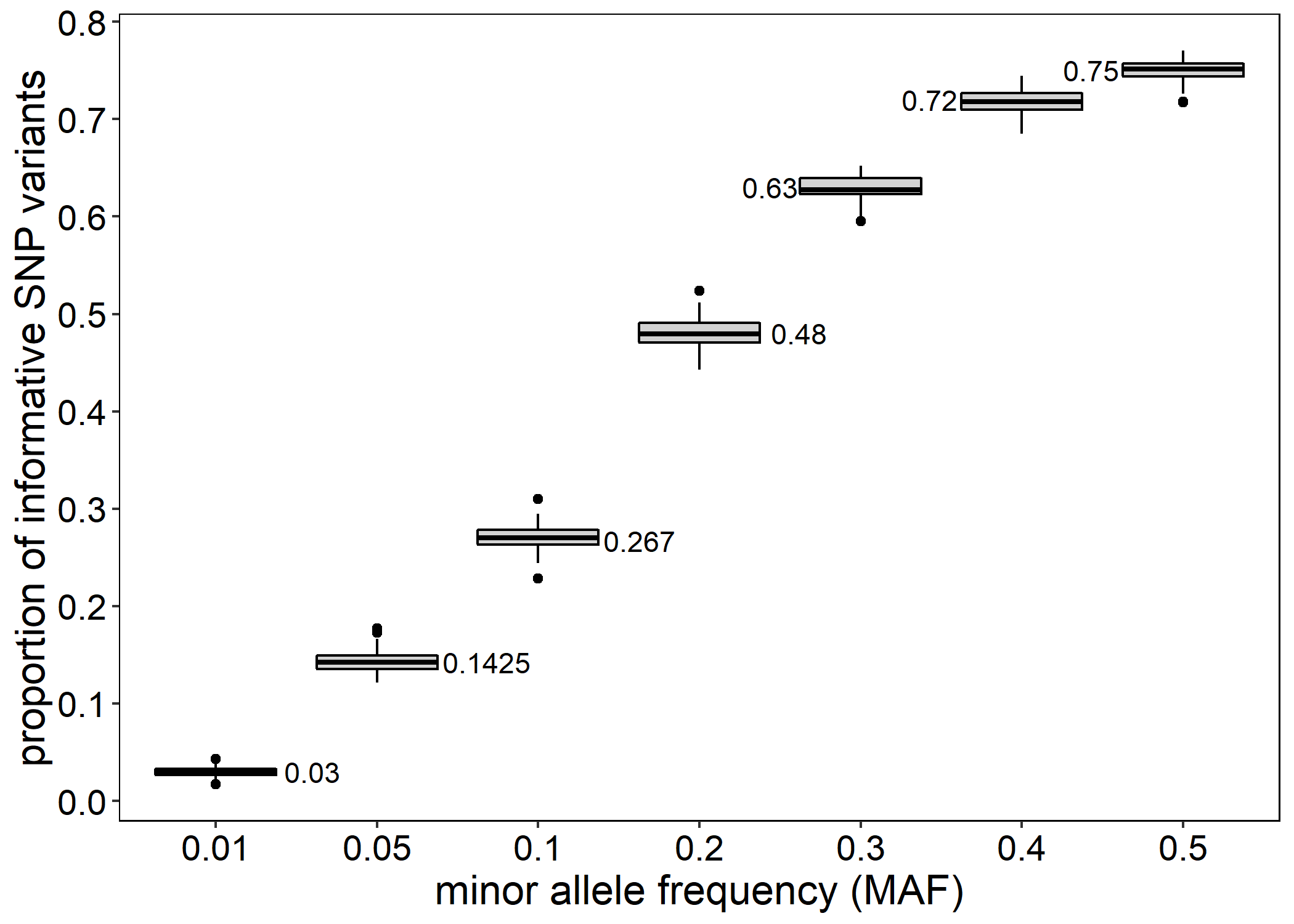

Supplement: S4 Fig — The proportion of informative variants (y-axis) in case of MAF of 1, 5, 10, 20, 30, 40 and 50% (x-axis) in 100 simulations with each MAF are represented with the box plots. The theoretical proportion of informative variants is denoted next to corresponding box plots for each MAF. (TIF) [file pone.0209139.s004.tif]

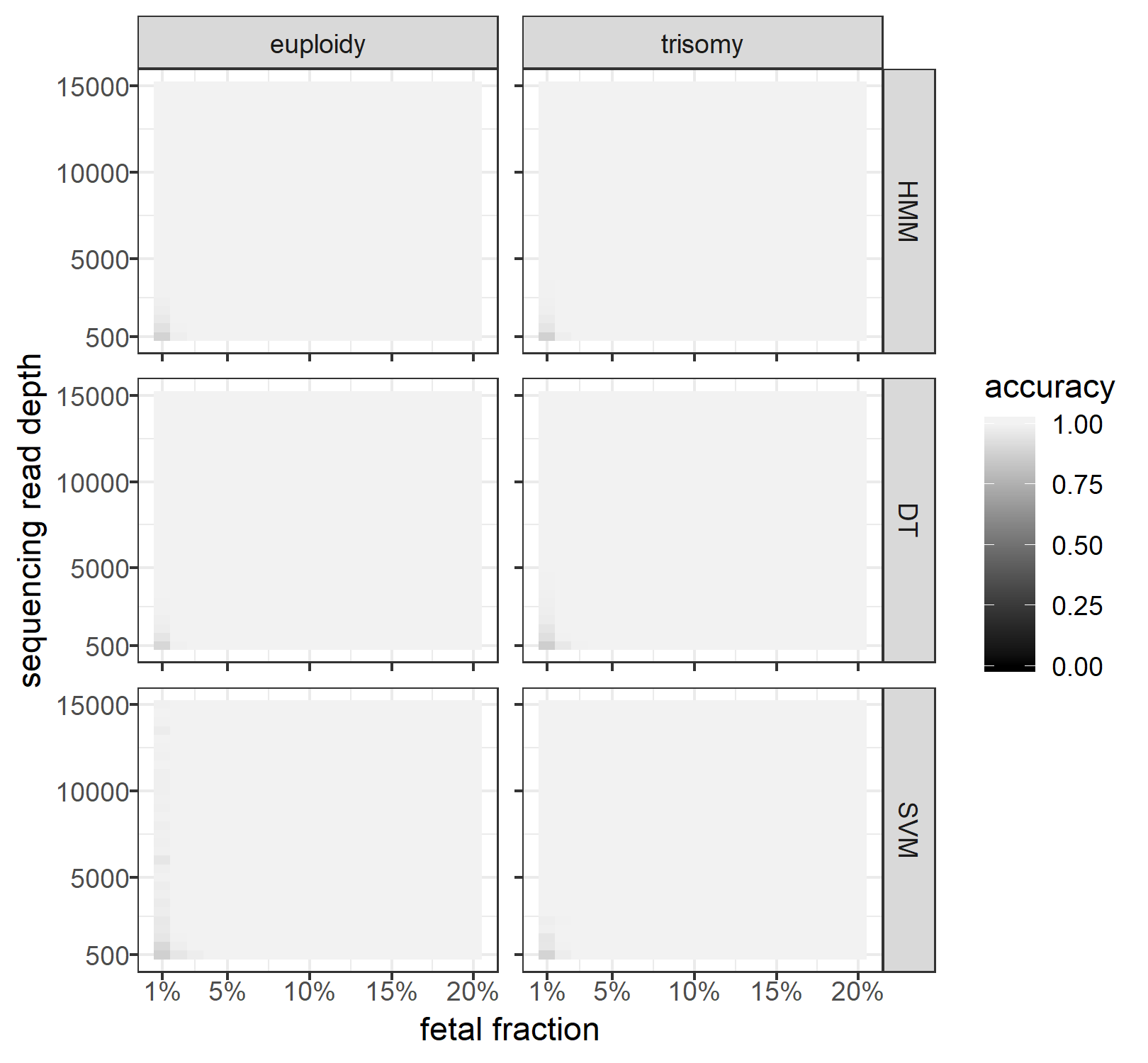

Supplement: S5 Fig — The simulated datasets of fetal euploidy and trisomy (vertical panels) were first classified by RC model and the resulting class frequencies were further classified by hidden Markov model (HMM) mode, decision tree (DT) and support vector machine (SVM) (horizontal panels). Each panel includes cells with different fetal DNA fractions (x-axis) and sequencing read coverages (y-axis). Each cell includes 10,000 cell-free DNA samples and the color represents the model classification accuracy. (TIF) [file pone.0209139.s005.tif]

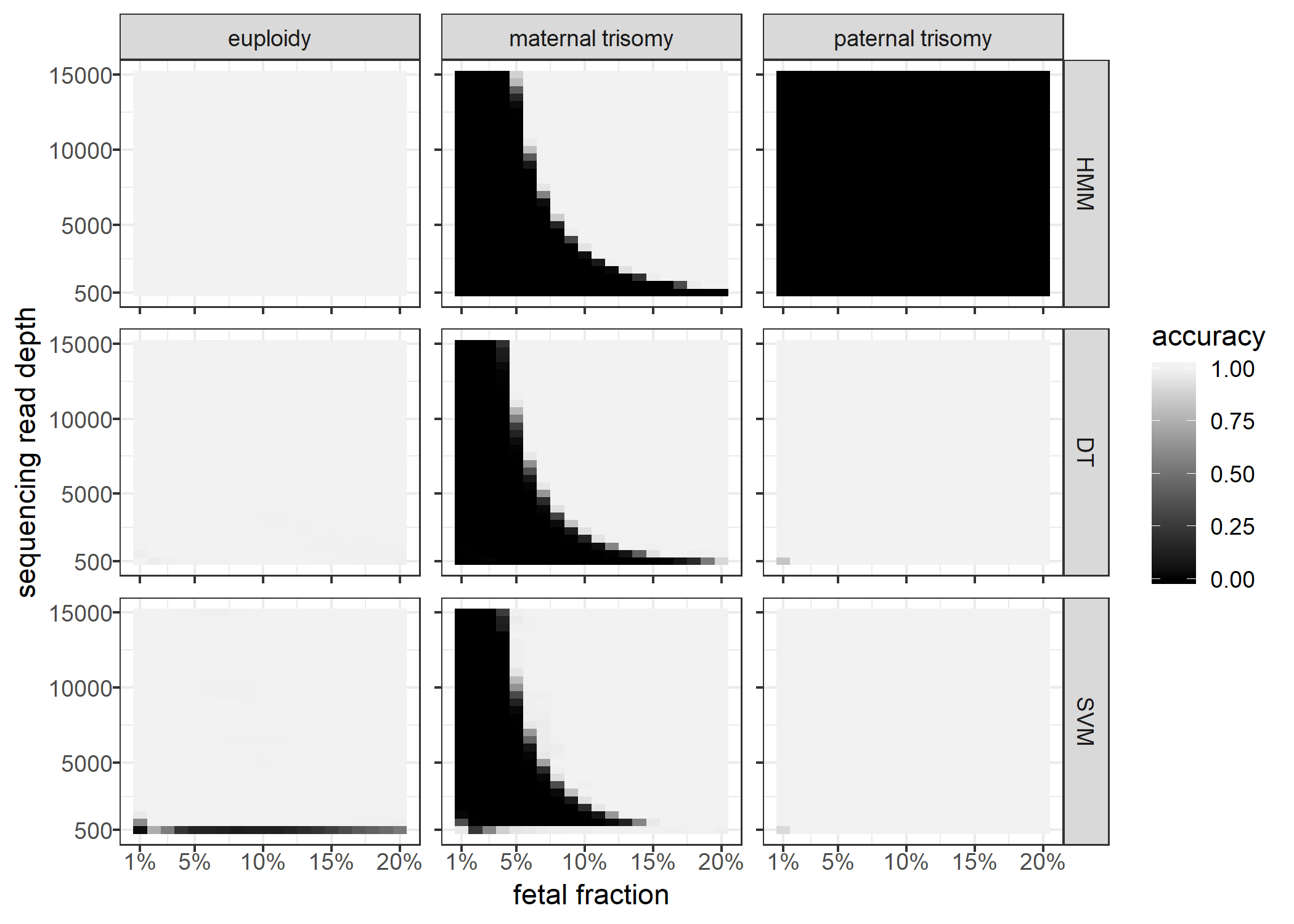

Supplement: S6 Fig — The simulated datasets of fetal euploidy, maternally and paternally trisomy (vertical panels) were first classified by the AR model and the resulting class frequencies were further classified by hidden Markov model (HMM) mode, decision tree (DT) and support vector machine (SVM) (horizontal panels). Each panel includes cells with different fetal DNA fractions (x-axis) and sequencing read coverages (y-axis). Each cell includes 10,000 cell-free DNA samples and the color represents the model classification accuracy. (TIF) [file pone.0209139.s006.tif]

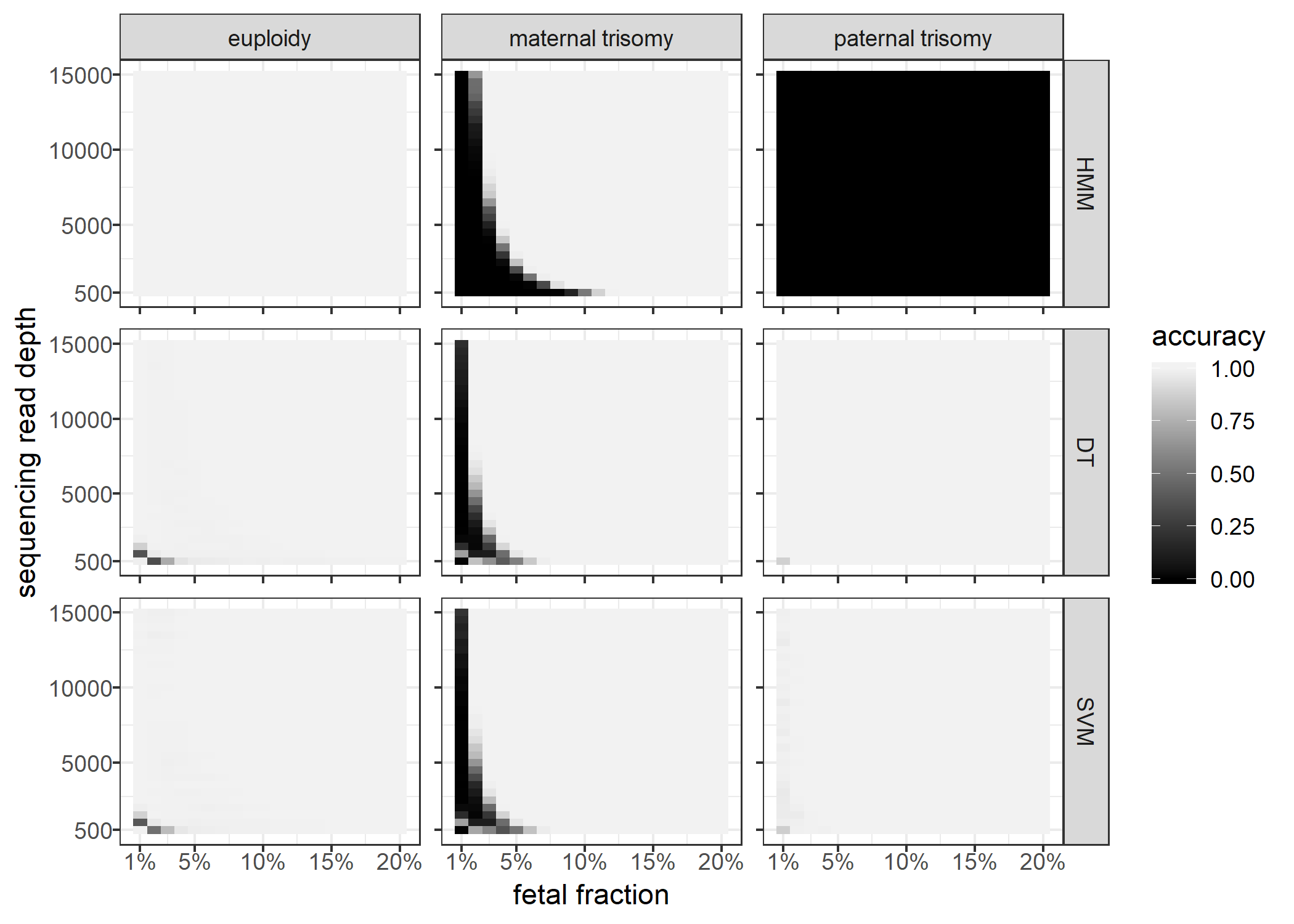

Supplement: S7 Fig — The simulated datasets of fetal euploidy, maternally and paternally trisomy (vertical panels) were first classified by RCAR model and resulting class frequencies were further classified by hidden Markov model (HMM) mode, decision tree (DT) and support vector machine (SVM) (horizontal panels). Each panel includes cells with different fetal DNA fractions (x-axis) and sequencing read coverages (y-axis). Each cell includes 10,000 cell-free DNA samples and the color represents the model classification accuracy. (TIF) [file pone.0209139.s007.tif]

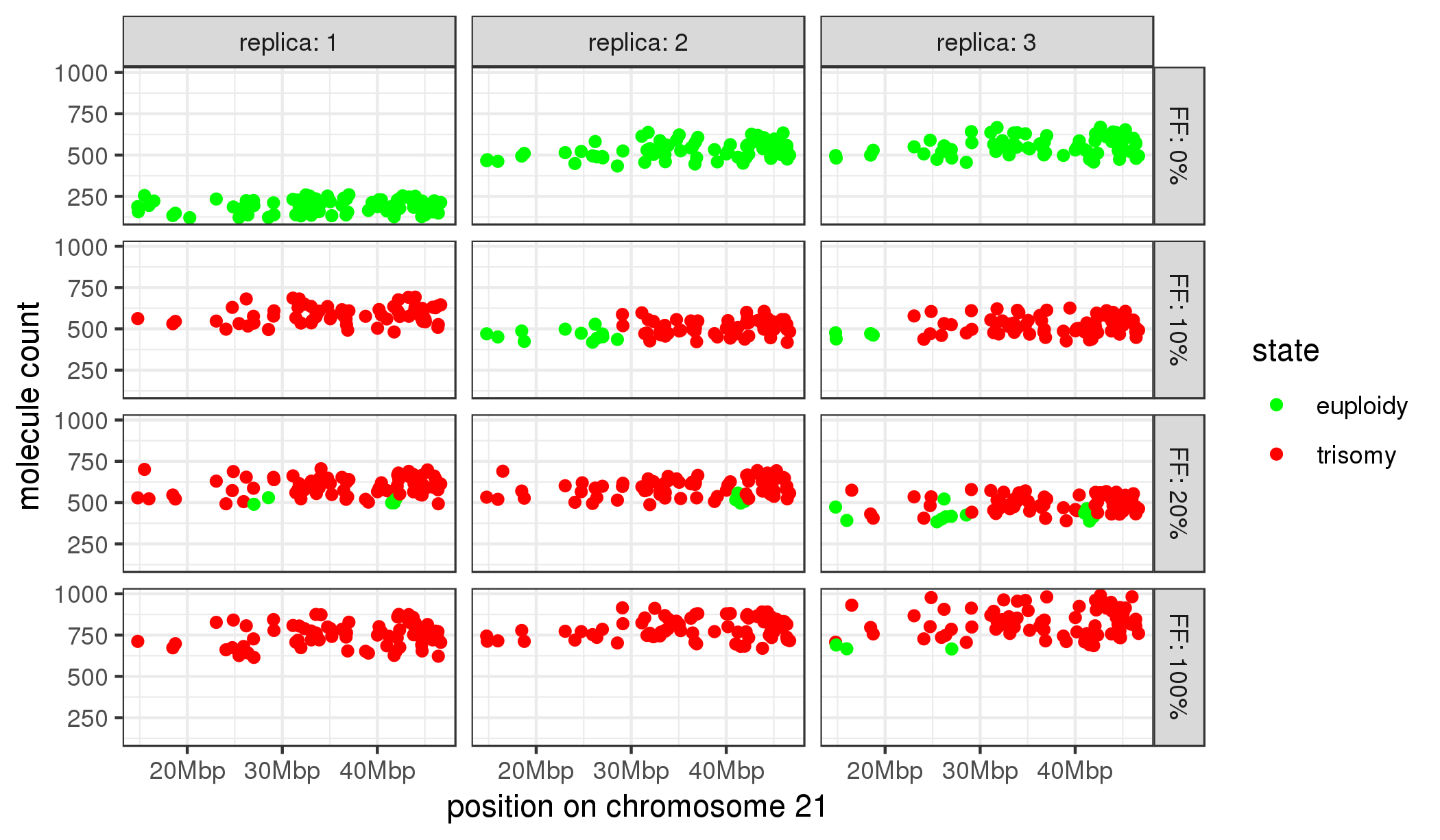

Supplement: S8 Fig — Experimentally controlled in vitro trisomy samples were created by mixing different proportions of genomic DNA from a non-trisomy 21 cell line with a genomic DNA from a trisomy 21 cell line to imitate different proportions of FF (horizontal panels), each in three replicas. Genomic DNA was sheared by sonication to mimic 160–180 bp cfDNA. DNA fragments were hybridized and sequenced using TAC-seq detector probes specifically designed to target only the reference chromosome 2 (68 targeted loci) and chromosome 3 (60 targeted loci), and the studied chromosome 21 (99 targeted loci). Read counts of targeted loci were converted to absolute molecular counts using unique molecular identifiers with the threshold of one to reduce the PCR amplification bias. Obtained absolute molecule counts of each sample were filtered by interquartile range to remove outliers and used as input to RC model to classify each targeted locus as “euploidy” (green color) or “trisomy” (red color). Sequentially classified targeted loci per sample are visualized as colored dots. (TIF) [file pone.0209139.s008.tif]
