# Supplementary material for: Computational framework for targeted high-coverage sequencing based NIPT
Source: PLoS One. 2019 Jul 8;14(7):e0209139. doi: 10.1371/journal.pone.0209139 (PMC6613673; doi:10.1371/journal.pone.0209139)
Supplement: S1 Table — Allelic ratio depends on fetal chromosomal condition, and maternal and fetal genotype. (DOCX) [file pone.0209139.s009.docx]

**S1 Table. Allelic patterns.** Allelic ratio depends on fetal chromosomal condition, and maternal and fetal genotype.

| Fetal condition | Maternal genotype | Fetal genotype | Major allele | Minor allele | Allelic ratio |
| --- | --- | --- | --- | --- | --- |
| Euploid | Homozygous | Homozygous | $2MA+2FA$ | - | - |
|  |  | Heterozygous | $2MA+FA$ | $FA$ | $\frac{2MA+FA}{FA}$ |
| Maternal trisomy |  | Homozygous | $2MA+3FA$ | - | - |
|  |  | Heterozygous | $2MA+2FA$ | $FA$ | $\frac{2MA+2FA}{FA}$ |
| Paternal trisomy |  | Homozygous | $2MA+3FA$ | - | - |
|  |  | Heterozygous | $2MA+2FA$ | $FA$ | $\frac{2MA+2FA}{FA}$ |
|  |  |  | $2MA+FA$ | $2FA$ | $\frac{2MA+FA}{2FA}$ |
| Euploid | Heterozygous | Homozygous | $MA+2FA$ | $MA$ | $\frac{MA+2FA}{MA}$ |
|  |  | Heterozygous | $MA+FA$ | $MA+FA$ | $\frac{MA+FA}{MA+FA}$ |
| Maternal trisomy |  | Heterozygous | $MA+2FA$ | $MA+FA$ | $\frac{MA+2FA}{MA+FA}$ |
| Paternal trisomy |  | Homozygous | $MA+3FA$ | $MA$ | $\frac{MA+3FA}{MA}$ |
|  |  | Heterozygous | $MA+2FA$ | $MA+FA$ | $\frac{MA+2FA}{MA+FA}$ |

*MA – maternal allele count; FA – fetal allele count*
