# Supplementary material for: Computational framework for targeted high-coverage sequencing based NIPT
Source: PLoS One. 2019 Jul 8;14(7):e0209139. doi: 10.1371/journal.pone.0209139 (PMC6613673; doi:10.1371/journal.pone.0209139)
Supplement: S2 Table — Each value represents an average classification accuracy over 1,200,000 simulated cell-free DNA samples with fetal (maternally or paternally inherited) trisomy at the different sequencing depth intervals (500–15,000 reads). (DOCX) [file pone.0209139.s010.docx]

**S2 Table. Fetal trisomy classification accuracy with different computational models and methods.** Each value represents an average classification accuracy over 1,200,000 simulated cell-free DNA samples with fetal (maternally or paternally inherited) trisomy at the different sequencing depth intervals (500 - 15,000 reads).

| Fetal fraction | Euploidy | | | | | | | | | | | | Trisomy | | | | | | Maternal trisomy | | | | | | Paternal trisomy | | | | | |
| --- | --- | --- | --- | --- | --- | --- | --- | --- | --- | --- | --- | --- | --- | --- | --- | --- | --- | --- | --- | --- | --- | --- | --- | --- | --- | --- | --- | --- | --- | --- |
|  | RC | | | RC (fixed FF) | | | AR | | | RCAR | | | RC | | | RC (fixed FF) | | | AR | | | RCAR | | | AR | | | RCAR | | |
|  | HMM | DT | SVM | HMM | DT | SVM | HMM | DT | SVM | HMM | DT | SVM | HMM | DT | SVM | HMM | DT | SVM | HMM | DT | SVM | HMM | DT | SVM | HMM | DT | SVM | HMM | DT | SVM |
| 1-5% | 1.00 | 1.00 | 1.00 | 1.00 | 0.90 | 0.98 | 1.00 | 1.00 | 0.98 | 1.00 | 0.99 | 0.99 | 1.00 | 1.00 | 1.00 | 0.11 | 0.96 | 0.92 | 0.02 | 0.07 | 0.10 | 0.47 | 0.73 | 0.73 | 0.00 | 1.00 | 1.00 | 0.00 | 1.00 | 1.00 |
| 6-10% | 1.00 | 1.00 | 1.00 | 1.00 | 1.00 | 0.99 | 1.00 | 1.00 | 0.97 | 1.00 | 1.00 | 1.00 | 1.00 | 1.00 | 1.00 | 1.00 | 1.00 | 1.00 | 0.63 | 0.75 | 0.78 | 0.96 | 1.00 | 1.00 | 0.00 | 1.00 | 1.00 | 0.00 | 1.00 | 1.00 |
| 11-15% | 1.00 | 1.00 | 1.00 | 1.00 | 1.00 | 1.00 | 1.00 | 1.00 | 0.97 | 1.00 | 1.00 | 1.00 | 1.00 | 1.00 | 1.00 | 1.00 | 1.00 | 1.00 | 0.89 | 0.93 | 0.97 | 1.00 | 1.00 | 1.00 | 0.00 | 1.00 | 1.00 | 0.00 | 1.00 | 1.00 |
| 16-20% | 1.00 | 1.00 | 1.00 | 1.00 | 1.00 | 1.00 | 1.00 | 1.00 | 0.98 | 1.00 | 1.00 | 1.00 | 1.00 | 1.00 | 1.00 | 1.00 | 1.00 | 1.00 | 0.96 | 0.98 | 1.00 | 1.00 | 1.00 | 1.00 | 0.00 | 1.00 | 1.00 | 0.00 | 1.00 | 1.00 |
| total | 1.00 | 1.00 | 1.00 | 1.00 | 0.97 | 0.99 | 1.00 | 1.00 | 0.97 | 1.00 | 1.00 | 1.00 | 1.00 | 1.00 | 1.00 | 0.78 | 0.99 | 0.98 | 0.62 | 0.68 | 0.71 | 0.86 | 0.93 | 0.93 | 0.00 | 1.00 | 1.00 | 0.00 | 1.00 | 1.00 |

*RC – read count; AR – allelic ratio; RCAR – read count and allelic ratio; HMM – hidden Markov model; DT – decision tree; SVM – support vector machine; FF – fetal fraction*
